# Mindfulness and Psychological Flexibility are Inversely Associated with Caregiver Burden in Parkinson’s Disease

**DOI:** 10.3390/brainsci10020111

**Published:** 2020-02-20

**Authors:** Martin Klietz, Simon C. Drexel, Theresa Schnur, Florian Lange, Adrian Groh, Lejla Paracka, Stephan Greten, Dirk Dressler, Günter U. Höglinger, Florian Wegner

**Affiliations:** 1 Department of Neurology, Hannover Medical School, Carl-Neuberg-Straße 1, 30625 Hannover, Germany; Simon.C.Drexel@stud.mh-hannover.de (S.C.D.); Theresa.Schnur@stud.mh-hannover.de (T.S.); Paracka.Lejla@mh-hannover.de (L.P.); Greten.Stephan@mh-hannover.de (S.G.); Dressler.Dirk@mh-hannover.de (D.D.); Hoeglinger.Guenter@mh-hannover.de (G.U.H.); Wegner.Florian@mh-hannover.de (F.W.); 2 Behavioral Engineering Research Group, KU Leuven, Naamsestraat 69, 3000 Leuven, Belgium; florian.lange@kuleuven.be; 3 Department of Psychiatry, Social Psychiatry and Psychotherapy, Hannover Medical School, Carl-Neuberg-Straße 1, 30625 Hannover, Germany; Groh.Adrian@mh-hannover.de

**Keywords:** Parkinson’s disease, caregiver burden, mindfulness, psychological flexibility, health-related quality of life

## Abstract

Parkinson’s disease (PD) is a neurodegenerative movement disorder with progressive impairments in activities of daily living. With disease progression, people with PD (PwP) need more help and care from their spouses or professional caregivers. Identifying factors that help caregivers to cope with their burden is needed to frame future interventions for PwP caregivers. Mindfulness and psychological flexibility might be factors contributing to resilience against the burden of giving care. In this cross-sectional questionnaire-based study, 118 PwP and their respective primary caregivers were included. Caregivers reported moderate burden and only mild depressive symptoms. Mindfulness measured by the Mindfulness Attention and Awareness scale (*p* 0.003) and psychological flexibility measured by Acceptance and Actions Questionnaire II (*p* 0.001) correlated negatively with caregiver burden. Data from this study indicate mindfulness and psychological flexibility are factors contributing to resilience against caregiver burden. Future interventions to reduce burden in PwP caregivers might be improved by the inclusion of mindfulness training programs.

## 1. Introduction

Parkinson’s disease (PD) is a common neurodegenerative disorder in the elderly, with a prevalence of more than 400,000 affected people in Germany [1]. People with Parkinson’s disease (PwP) suffer from typical motor and non-motor symptoms [2,3]. With progression of the disease, PwP become increasingly care-dependent and lose their autonomy [4]. Interestingly, this time frame of autonomy loss and accumulation of symptoms until death is very long in Parkinson’s disease, because PwP only have a slightly increased mortality [5,6,7]. Therefore, PwP suffer a long time from profoundly reduced quality of life. During this period, related caregivers help the patient for increasing amounts of time. In the late phase of the disease, caregivers experience multidimensional adverse effects, resulting in caregiver burden [8,9]. Increasing burden may even lead to caregiver burnout and institutionalization of the patient in a residential home [10]. In addition, clinical depression may occur [11]. In Germany, attention has recently been given to the situation of caregivers of PwP [11,12]. The detection of preventive factors that reduce the development of caregiver burden is desperately needed to lessen the burden that many relatives of PwP feel (see Mosley et al., 2017 [9] for a review of currently investigated interventions).

Mindfulness is defined as the tendency to purposely bring one’s attention to experiences occurring in the present moment without judgement [13]. Psychological flexibility refers to the extent to which a person can cope with changing circumstances and think about problems and tasks in novel and creative ways [14]. Mindfulness and mindfulness-based interventions have found their way into the treatment of mood and anxiety disorders [15]. Recently, psychological flexibility and mindfulness have been discussed as protective personality traits for the prevention or reduction of caregiver burden [16,17,18]. Mindfulness and psychological flexibility have been found to be related constructs that nonetheless reflect a substantial amount of non-overlapping information. Both constructs appear to explain unique interindividual variations in psychological health [19].

This study aimed to investigate the role of mindfulness and psychological flexibility as preventive intrinsic factors for caregivers of PwP to reduce caregiver burden in a cross-sectional questionnaire-based fashion. We hypothesized that caregivers with high mindfulness and psychological flexibility are less burdened by giving care to a spouse suffering from Parkinson’s disease. After confirmation of the positive influence of mindfulness and psychological flexibility, specific training programs could be integrated to interventions in order to reduce caregiver burden in caregivers of PwP.

## 2. Materials and Methods

### 2.1. Procedures

Approval from the local Ethics Committee of Hannover Medical School was obtained (No. 3178-2016, Amendment in 2018) and patients, as well as their caregivers, gave written informed consent. The research was conducted ethically in accordance with the World Medical Association Declaration of Helsinki. PwP and their caregivers were recruited by newspaper ads, via the German PD association and the Hilde-Ulrichs-Foundation. Participants received the study questionnaires via mail and were asked to return the completed forms by mail with postage fees covered by the study center. In total, we sent 191 questionnaires to participants; 118 of these were received back at the study center, representing a response rate of 61.8%.

### 2.2. Participants

Patients with neurologically confirmed PD for at least one year were included in the study. Patients suffering from atypical Parkinsonism were excluded from the study. In the case of multiple caregivers, only the primary caregiver (mostly spouses) of the PD patient was included. Professional caregivers were not included in our study because of their different coping strategies compared with informal caregivers.

### 2.3. Measures

Caregivers were asked to complete a recently developed and validated German version of the Parkinson’s disease caregiver burden questionnaire (PDCB) [12,20]. In brief, the PDCB includes 20 items on a five-point Likert scale (0 to 4). Participants can reach a maximum of 80 points on this part of the questionnaire. In addition, participants had to rate their global burden as a caregiver on a scale from 0 to 100 in the second part of the questionnaire. Finally, points from the first part and 20% of the global burden of part two were summed up for the total caregiver burden. A maximal score of 100 can be reached as total score, indicating the highest burden [20]. In the English version of the PDCB, Cronbach’s alpha was 0.856 and the PDCB correlated significantly with the CBI as a general caregiver burden questionnaire [20]. In the validated German version of the PDCB, the items showed a Cronbach’s alpha score of 0.80 and a significant correlation with the ZBI-22 (r 0.71; *p* < 0.001 as general caregiver questionnaire and total SF-36 score of the caregiver r-0.40; *p* 0.001) [8,12].

Caregiver and PwP depression was measured by Beck’s Depression Inventory (BDI) [21]. The BDI is one of the standard measures for depressive symptoms with good reliability quantified in various studies (for review, see [22]).

In addition, the health-related quality of life (HR-QoL) of the caregivers was assessed by the Short-Form 36 Health Survey (SF-36) [23]. The SF-36 is one of the gold standard forms for the assessment of health-related quality of life with good reliability of the items in several studies with a Cronbach’s alpha > 0.80 and reliability compared to other measures of quality of life of >0.90 [24]. We calculated an average score across the subscales to analyze overall health-related quality of life more generally, as was also done in other recent publications [12,25,26].

Caregivers and PwP were also asked to provide general information about their background and demographics (e.g., patients’ disease duration, education of both PwP and caregiver (as done in [27]), relationship, as well as the daily amount of time dedicated to giving care to the patient).

To obtain an indicator of patients’ PD-related impairment, PwP were asked to complete the Movement Disorders Society Unified Parkinson’s Disease Rating Scale (MDS-UPDRS) II as a PD-specific measure of motor impairment in daily living [28,29]. To avoid anosognosia, PwP suffering from cognitive impairments were asked to complete the scales with the help of their caregiver (as done in [4,12]).

To assess health-related quality of life, PwP were provided with a copy of the Parkinson’s Disease Quality of Life Questionnaire 8 (PDQ-8) [30]. The PDQ-8 is the short version of the PDQ-39, including eight dimensions of HR-QoL [31]. PDQ-8 shows a high internal consistency with a Cronbach’s alpha of 0.59–0.79. This has been confirmed in more recent studies [32].

For the measurement of caregiver mindfulness, the Mindfulness Attention and Awareness Scale (MAAS) was used. The MAAS is a single-factor measure for mindfulness containing 15 items. It can be answered on a six-point Likert scale with a range from 1 to 6. Higher scores indicate more mindfulness. A minimal score of 15 and a maximal score of 90 points can be reached [33]. The scale shows good internal reliability with a Cronbach’s alpha of 0.89 [34,35]. The scale correlates significantly with health complaints and satisfaction in life of Norwegian adults [36].

The Acceptance and Actions Questionnaire II (AAQ-II) is a one-factor measure for psychological flexibility and includes seven items, which can be answered on a six-point Likert scale ranging from 1 to 6. Higher scores indicate a greater extent of psychological inflexibility. A minimal score of 7 and a maximal score of 42 can be reached [14]. The scale shows a high internal consistency with a Cronbach’s alpha of 0.84 and good test–retest reliability 0.81 [14]. These results could be confirmed in several large samples [34].

### 2.4. Analyses

Data were displayed by mean, standard deviation, minimal, and maximal values. For linear regression analyses, the level of significance was set to 0.05. To correct for multiple testing, the *p*-value was adjusted to the number of analyses (0.05/*n*). To avoid confounders or co-correlations, we included significant items from the linear regression analysis in a multiple regression model. Statistical analyses were carried out in SPSS^TM^ 25 (SPSS In., Chicago, IL, USA). The statistical analysis was counseled by the Institute of Biometrics, Hannover Medical School, Hannover, Germany.

## 3. Results

### 3.1. Characteristics of PwP and Their Caregivers

All demographic- and disease-related characteristics of PwP and caregivers are displayed in Table 1. PDCB as an outcome variable showed substantial variation as indicated by an SD of 27.8 points, min score of 0 and max score of 100 (Table 1). Recruited PwP were in all stages of PD and had a wide variability of disease duration and disease stages. Concerning the main interest of our study, caregivers revealed substantial variations in their measures of mindfulness and psychological flexibility. It thus seems unlikely that regression analysis is affected by variance restriction of our data. Detailed SF-36 subscales of the caregivers are displayed in Appendix A Table A1.

### 3.2. Variables Associated with Caregiver Burden in PwP

To test our hypotheses that mindfulness and psychological flexibility might be protective against the burden of the caregivers, PDCB was regressed on PD- and caregiver-related variables, including the MAAS and AAQ-II of the caregiver. MDS-UPDRS part II and PDQ-8 were patient-related factors correlating significantly with caregiver burden after correction for multiple comparisons. Caregiver factors SF-36, AAQ-II, and MAAS were also found to correlate significantly with the burden of the caregivers after correction for multiple comparisons (Table 2).

To test for co-correlations, we added all significant variables in a multiple regression analysis (Table 3). In the multiple regression analysis, only MDS-UPDRS part II and SF-36 of the caregivers were significantly associated with caregiver burden. MAAS and AAQ-II did not reach statistical significance. As indicated by low beta, both variables explained only minor parts of variance in the model. Hence, all variance inflation factors of all included variables were below 5, which is why we assume that that multicolinearity did not influence the quality of our multiple regression analysis.

## 4. Discussion

In this study, we investigated the correlation of mindfulness and psychological flexibility as a personality trait of the caregiver with burden of PD caregivers in a large German cohort. Both higher scores for mindfulness and psychological flexibility were associated with less-severe scores in the PDCB as a valid and specific measure for PD-specific caregiver burden [12]. It seems reasonable that both mindfulness and psychological flexibility represent preventive features for caregiver burden. In the presented cohort, mindfulness measured by the MAAS and psychological flexibility measured by AAQ-II questionnaires were very well distributed with sufficient levels of variance to detect robust correlations. However, the effect size of these two personality traits seems relatively low compared with disease-specific factors like PD symptoms, patient quality-of-life restrictions, caregiver quality-of-life restrictions, and depressive symptoms of the caregiver [11,37,38,39].

PwP suffer from a large variety of motor and non-motor symptoms [4,40,41]. These symptoms clearly have an impact on the HR-QoL of the patients and further on caregiver burden [2,42,43,44,45,46,47,48]. Cognitive symptoms of PwP are correlated with a worse extent of caregiver burden [49,50]. Particularly, attention and language deficits of PwP seem to be associated with caregiver burden [26]. Disorders of impulse control affect caregiver burden in advanced PD patients [10]. Additionally, motor symptoms of PwP quantified by the MDS-UPDRS part II, MDS-UPDRS part III, or Barthel Index have been proven to affect the burden of the caregivers [39,47,51].

Mindfulness refers to the tendency to purposely bring one’s attention to experiences occurring in the present moment with curiosity but without emotional judgement [13]. In view of our data, this tendency may relate to some degree of protection against caregiver burden. Mindfulness could help to improve coping and reduce stress of the caregivers. In PD, the caregiving situation becomes increasingly stressful with disease progression [4,44,47,50,52] and mindfulness might be particularly helpful during later stages of the disease. Additionally, mindfulness may help to mitigate caregivers’ tendency to overcommit in the caregiver role [53,54,55,56].

Mindfulness interventions have already shown some positive effects in PwP [17,18]. Data from interventions in PwP caregivers are lacking, but high-quality evidence is available on the effect of mindfulness-based interventions on professionals’ mental health [57].

Results for mindfulness-related interventions with the aim of increasing mindfulness in caregivers for patients with dementia reported a large effect on reduction of depressive symptoms and a moderate effect on the reduction of perceived burden of the caregivers [58]. The most established intervention to increase mindfulness is the mindfulness-based stress reduction (MBSR) training of Kabat-Zinn et al. [59], but many alternative validated MBSR interventions are available [39,40]. In the future, mindfulness-related interventions could represent a promising treatment to reduce caregiver burden [60].

With the progression of PD, the psychological flexibility of the patients decreases [61,62,63]. At the same time, the caregivers’ tasks increase, because the patient suffers from more severe motor and non-motor symptoms. Therefore, the caregiver must help the patient in more situations of daily living, resulting in elevated burden, which may lead to caregiver depression. In our study, caregivers with increased psychological flexibility suffered from less burden, indicating psychological flexibility could be a preventive factor for caregiver burden. In a recent review by Whiting et al., psychological flexibility seems to be associated with positive response to therapies and less self-reported distress in patients with traumatic brain injury [64]. Lamothe et al. confirmed in a systematic meta-analysis that MBSR and MBSR-based interventions contribute to professionals’ mental health [57].

A limitation of this study is that we only reported outcomes in a cross-sectional manner, which did not allow us to determine a causal relationship between mindfulness and psychological flexibility with caregiver burden. Longitudinal data would be desirable to see long-term effects of mindfulness and psychological flexibility on caregiver burden and caregivers’ health-related quality of life.

Further, a selection bias in the recruitment cannot be ruled out, since extremely burdened caregivers often did not reply and caregivers with more interest in mindfulness were more likely to participate in this study. Finally, this study did not aim to specifically investigate the relationship of non-motor symptoms to caregiver burden. This is why our study design did not include measures of non-motor symptoms, such as MDS-UPDRS part I, Non-Motor Symptoms Questionnaire, or other clinical scales for specific non-motor symptoms like cognitive testing, hallucinations, or disorders of impulse control [10,38,50].

## 5. Conclusions

This is one of the first studies reporting clues for possible preventive properties of mindfulness and psychological flexibility in the context of the perceived burden of PD caregivers. Future interventions for caregiver burden may include mindfulness and psychological flexibility trainings to help PD caregivers to increase these personality traits in order to cope better with the burdensome situation. Further longitudinal studies are needed to clarify the impact of mindfulness and psychological flexibility as individual personality traits on prevention of caregiver burden for caregivers of PwP.

## Figures and Tables

**Table 1:** Characteristics of people with Parkinson’s disease (PwP, *n* = 118, 45 females) and caregivers (*n* = 118, 78 females).

	Mean ± SD	Min	Min
**PwP**			
Age (years)	68.7 ± 10.1	40	87
Disease duration (years)	12.0 ± 7.5	1	37
Hoehn and Yahr stage	2.9 ± 1.1	1	5
MDS-UPDRS part II	19.0 ± 11.7	1	47
PDQ-8	40.4 ± 18.0	3.1	81.3
BDI	12.6 ± 8.1	0	39
Education level	low 4.4%, middle 57.5%, high 38.1%		
**Caregivers**			
Age (years)	65.4 ± 11.0	19	87
Caregiving hours/d	5.8 ± 6.4	0	24
PDCB	37.1 ± 27.8	0	100
SF-36	58.8 ± 17.8	5.8	85.6
BDI	9.8 ± 7.0	0	36
AAQ-II	17.2 ± 8.2	7	39
MAAS	66.4 ± 12.1	37	90
Education level	low 6.1%, middle 59.1%, high 34.8%		
Relationship to PwP	Spouse 93.2%, Child 6.8%,		

BDI: Beck’s Depression Inventory; AAQ-II: Acceptance and Actions Questionnaire II; MAAS: Mindfulness Attention Assessment Scale; MDS-UPDRS: Movement Disorders Society Unified Parkinson’s Disease Rating Scale; PDCB: Parkinson’s disease caregiver burden inventory; PDQ-8: Parkinson’s Disease Questionnaire 8; SF-36: WHO Short-Form 36 Health Survey; SD: standard deviation.

**Table 2:** Linear regression analysis of factors associated with caregiver burden measured by PDCB.

Variables	*R* ^2^	Beta	*p*
PwP age	0.0048	0.069	0.475
PwP sex (m = 0, f = 1)	0.0400	−0.200	0.034*
PwP education level (1 = low, 2 = middle, 3 = high)	0.0017	−0.041	0.669
PwP disease duration	0.0858	0.293	**0.002** **
MDS-UPDRS part II	**0.1892**	**0.439**	**<0.001** **
PDQ-8	**0.1789**	**0.425**	**<0.001** **
BDI of PD patients	0.0671	0.175	0.069
Caregiver age	0.0061	0.078	0.413
Caregiver sex (m = 0, f = 1)	0.0199	0.141	0.138
Caregiver educational level (1 = low, 2 = middle, 3 = high)	0.0050	−0.071	0.462
Caregiving hours per day	0.0790	0.272	0.007 *
SF-36 of caregivers	**0.0973**	**−0.348**	**<0.001** **
BDI of caregivers	0.0586	0.242	0.011 *
AAQ-II of caregivers	**0.1163**	**0.326**	**0.001** **
MAAS of caregivers	**0.0807**	**−0.282**	**0.003** **

Linear regression analysis of factors contributing to caregiver burden. *p*-Value adjustment for multiple comparisons was 0.05/15. * Indicates significant correlations at *p* < 0.05; ** indicate significance level at *p* < 0.0033, printed in bold.

**Table 3:** Multiple regression analysis of significant factors associated with caregiver burden measured by PDCB in linear regression analysis.

	B (95% CI)	Beta	*t*	*p*	VIF
(Constant)	27.712 (−13.901; 68.325)		1.324	0.189	
PwP Sex (m = 0, f = 1)	−8.697 (−17.622; 0.229)	−0.162	−1.937	0.056	1.180
PwP disease duration	0.169 (−0.479; 0.816)	0.048	0.518	0.606	1.482
MDS-UPDRS part II	0.608 (0.011; 1.205)	0.265	2.023	**0.046**	2.907
PDQ-8	0.862 (−0.126; 1.849)	0.214	1.735	0.086	2.577
SF-36 of caregivers	−0.367 (−0.655; −0.078)	−0.253	−2.525	**0.013**	1.698
AAQ-II of caregivers	0.301 (−0.338; 0.940)	0.094	0.938	0.351	1.710
MAAS of caregivers	−0.043 (−0.441; 0.356)	−0.020	−0.213	0.832	1.473

Multiple regression analysis of significant factors associated with caregiver burden measured by PDCB in linear regression analysis. Adjusted *R*^2^ 0.451. *p* < 0.05 was considered as significant. VIF, variance inflation factor.

## Appendix A

**Table A1:** Detailed SF-36 subscores of the caregivers (*n* = 118).

	Mean ± SD	Min	Min
SF-36 total	58.8 ± 17.8	5.8	85.6
SF-36 physical functioning	79.5 ± 24.0	0	100
SF-36 physical role functioning	66.4 ± 38.7	0	100
SF-36 bodily pain	66.2 ± 27.3	0	100
SF-36 general health perception	52.8 ± 10.6	30	100
SF-36 vitality	54.6 ± 20.8	0	100
SF-36 social role functioning	78.1 ± 24.0	0	100
SF-36 emotional role functioning	66.1 ± 35.7	0	100
SF-36 emotional role functioning	66.3 ± 18.5	12.0	100
SF-36 mental health	58.0 ± 28.0	20.0	100
